# Dietary Sodium Butyrate Changed Intestinal Histology and Microbiota of Rainbow Trout (*Oncorhynchus mykiss*), but Did Not Promote Growth and Nutrient Utilization

**DOI:** 10.1155/2023/3706109

**Published:** 2023-01-07

**Authors:** Xia Lin, Chunyan Zhang, Kailin Cao, Zhendong Li, Zhongshen Zhao, Xiaoqin Li, Xiangjun Leng

**Affiliations:** ^1^National Demonstration Center for Experimental Fisheries Science Education, Shanghai Ocean University, Shanghai 201306, China; ^2^Shanghai Ocean University, Centre for Research on Environmental Ecology and Fish Nutrition of Ministry of Agriculture, Shanghai 201306, China; ^3^Zhumadian Huazhong Chia Tai Co., Ltd., Henan 463023, China

## Abstract

The study investigated the effects of dietary sodium butyrate (SB) on the growth performance, nutrient utilization, intestinal histology, and microbiota of rainbow trout (*Oncorhynchus mykiss*). A high fishmeal diet and a low fishmeal diet were formulated to contain 200 g/kg or 100 g/kg fishmeal, respectively. Coated SB (50%) was supplemented to each of the diets at levels of 0, 1.0, and 2.0 g/kg to create 6 diets. The diets were fed to rainbow trout with initial body weight of 29.9 ± 0.2 g for 8 weeks. Compared to the high fishmeal group, the low fishmeal group showed significantly lower weight gain (WG), intestine muscle thickness, and significantly higher feed conversion ratio (FCR) and amylase activity (*P* < 0.05). The supplementation of SB in high or low fishmeal diet did not significantly affect the WG, FCR, protein retention, and the digestibility of dry matter and crude protein (*P* > 0.05). The supplementation of 2.0 g/kg SB in low fishmeal diet significantly increased the villus height, villus width, and muscular thickness, while the supplementation of 2.0 g/kg SB in high fishmeal diet also significantly increased the intestinal villus height (*P* < 0.05). In intestinal microbiota, the supplementation of 2.0 g/kg SB significantly increased the abundance of *Proteobacteria* and *Aeromonas*, and decreased the abundance of *Firmicutes* and *Mycoplasma* (*P* < 0.05), but the flora at genus and phylum level were not affected by SB supplementation in low fishmeal diet (*P* > 0.05). In conclusion, the addition of SB in diets containing 100 or 200 g/kg fishmeal did not enhance the growth performance and nutrient utilization of rainbow trout, but improved intestinal morphology and changed intestinal microbial flora.

## 1. Introduction

Rainbow trout (*Oncorhynchus mykiss*) is an important cold water aquaculture fish in the world. In China, the aquaculture production of rainbow trout reached 37,841 tons in 2020 [1]. Rainbow trout is a carnivorous fish, and the commercial diets usually contain high amount of fishmeal. Due to the scarcity of resource and rising price of fishmeal, reducing the inclusion level of fishmeal in aquafeeds, especially in carnivorous fish diets, has become a hot spot in aquafeed industry [2]. Generally, the high replacement of fishmeal with plant protein sources leads to the decrease of growth performance, feed intake, and digestibility [3] in aquaculture fishes due to the presence of antinutritional factors and deficiencies in some essential amino acids, as well as the damage to physical barrier of the intestine and inflammatory reactions [4]. Therefore, the application of functional nutrients may be an effective way to improve the utilization efficiency of nonfishmeal protein sources.

Sodium butyrate is the sodium salt of butyric acid, and the active ingredient is butyric acid. Previous studies have shown that butyric acid is the direct source of energy for intestinal epithelial cells [5], which can promote intestinal cell proliferation, improve intestinal morphology, increase the digestion and absorption of nutrients, and improve animal growth [6, 7]. With its hydrophilic and lipophilic properties, butyric acid can inhibit harmful bacteria and promote the reproduction of beneficial bacteria after entering bacterial cells, thus maintaining intestinal health. The dietary addition of 0.3% sodium butyrate product (70% effective content) increased the specific growth rate of sea bream (*Sparus aurata*) by 5% and decreased feed conversion ratio by 8% [8]. [9]) found that golden pompano (*Trachinotus ovatus*) juvenile presented the highest weight gain and specific growth rate when the fish was fed a diet containing 2.0 g/kg sodium butyrate. In black bream (*Acanthopagrus schlegelii*) juvenile, the dietary addition of 0.2% microencapsulated sodium butyrate increased weight gain and feed efficiency [10]. Abd El-Naby et al. [11] and Dawood et al. [12] reported that the addition of sodium butyrate improved the growth performance and immunity of tilapia (*Oreochromis mossambicus*). Meanwhile, sodium butyrate supplementation was reported to maintain the intestinal health of tilapia [13]. In addition, dietary sodium butyrate also promoted the growth and improved intestinal function of grass carp (*Ctenopharyngodon idella*) juveniles [14–16], turbot (*Scophthalmus maximus*) [17], giant bony tongues (*Arapaima gigas*) [18], and yellow drum (*Nibea albiflora*) [19].

The previous studies have shown the beneficial effects of sodium butyrate in some fishes, but few studies have been conducted in salmonids, with only a few reports using sodium butyrate as part of a mixture of organic salts. Gao et al. [20] found that the dietary addition of 10 g/kg of a mixture of sodium formate and sodium butyrate did not significantly affect the growth and feed utilization of rainbow trout. In Atlantic salmon (*Salmo salar*), dietary supplementation of short-chain fatty acid (salt) mixtures (0.5% and 2.0%) (acetic acid: propionic acid: sodium butyrate = 5 : 5 : 2) also showed no significant effects on growth performance, apparent nutrient digestibility, total lipid, and fatty acid composition of fish and intestinal tissues [21]. From the above studies, it is still unclear if salmonids can effectively utilize sodium butyrate or not. Therefore, the present study aimed to investigate the effects of different levels of sodium butyrate supplementation in diets on growth performance, nutrient utilization, intestinal histology, and microbiota of rainbow trout. The results will guide the application of sodium butyrate in aquafeeds.

## 2. Materials and Methods

### 2.1. Ethical Statement

The procedures have been authorized by the Animal Ethics Committee for Experiments on Animals of Shanghai Ocean University and implemented in accordance with the experiment animal welfare regulation formulated by the Chinese Association for Laboratory Animal Science.

### 2.2. Experimental Diets and Design

A two-factor trial was designed with two levels of fishmeal and three levels of sodium butyrate supplementation. Firstly, a high fishmeal diet was formulated to contain 200 g/kg of fishmeal, and then, 100 g/kg of fishmeal was replaced by soybean meal and other proteins to form a low fishmeal diet with 100 g/kg of fishmeal inclusion. Based on the above two diets, 0, 0.1, and 0.2 g/kg of coated sodium butyrate (effective content 50%, provided by Zhumadian Huazhong Chia Tai Co., Ltd.) were added to form six isonitrogenous and isoenergetic diets. The protein ingredients were crushed and sieved through 60 mesh (diameter of 0.25 mm), and then, all the ingredients including 0.5 g/kg yttrium oxide were mixed and extruded to form sinking pellets of 3.0 mm diameter by single screw extruder (LX-75 Extruder, Longxiang Food Machinery Factory, Longyao, China). The extruding temperature was (85 ± 5) °C, and drying temperature was 50°C. After cooling, all diets were sealed and stored at 4°C until use. Powdered sodium butyrate mixed with other ingredients was added to the diets. Yttrium oxide was added in all diets as an inert marker to determine the apparent digestibility of nutrients. The formulation and proximate composition of experimental diets are shown in Table 1.

### 2.3. Fish and Feeding Management

Rainbow trout were purchased from Ruominghao Aquaculture Co., Ltd. (Kunming, China). After 2 weeks of adaptation period, all fish were fasted for 24 hours, and then, 396 fish with average body weight of 29.90 ± 0.20 g were randomly divided to 18 tanks with 6 treatments, 3 replicates per treatment, and 22 fish per replicate. The feeding trial was conducted in 18 circular tanks with a diameter of 1.0 m and a depth of 0.8 m (water volume of 650 L). During the feeding period, the fish were fed twice a day (8 : 30 and 15 : 30) with feeding rate of 3%-5% of body weight, which was adjusted appropriately according to the feeding behavior and weather conditions to ensure no diet residue in 2 min after feeding. The feed intake of each tank was kept with the similar amount. The feces at the bottom of the tank were siphoned out in 2 hours after feeding. The water was renewed about 1/3 every three days to ensure the water quality. During the feeding period, the water temperature (12-16°C), dissolved oxygen (6-7 mg/L), pH (7.2-7.6), ammonia nitrogen (≤0.2 mg/L), and nitrite (≤0.1 mg/L) were measured daily. The feeding trial was performed in the indoor aquaculture system at Binhai Aquaculture Station of Shanghai Ocean University for 8 weeks.

### 2.4. Sample Collection

Before the feeding trial, ten fish were collected and stored at -20°C for the initial whole body composition analysis. At the end of the feeding trial, all fish were fasted for 24 hours, and the fish in each tank were counted and weighed. Nine fish were randomly selected and anesthetized from each tank. Among these fish, three fish were stored at -20°C for the proximate composition analysis of the whole body. Another three fish were measured for body length and body weight, and then, blood was drawn from caudal vein and centrifuged for 10 min at 4000 r/min and 4°C. The serum was collected and stored at -80°C for the determination of biochemical indexes. After blooding, the visceral mass and liver were isolated and weighed, and then, the intestine was isolated, and a part of foregut (1–2 cm) was sampled and preserved in Bouin's solution after removing mesenteric adipose tissue. For the remaining three fish, the hindgut was collected under aseptic conditions after peeling off mesenteric adipose tissue and removing chyme and then stored in liquid nitrogen for intestinal microbiological analysis. Starting from the 5th week of the feeding trial, the intact feces was collected daily by siphoning and stored at -20°C for the digestibility measurements.

### 2.5. Measurement Indicators and Methods

#### 2.5.1. Growth Performance and Body Indices

Weight gain (WG), feed conversion ratio (FCR), survival rate (SR), viscerosomatic index (VSI), hepatosomatic index (HSI), and condition factor (*K*) were calculated as follows:

WG(%) = 100 × (FBW − IBW)/IBW,

FCR = *W*_*f*_/(FBW − IBW),

SR(%) = 100 × *N*_*t*_/*N*_0_,

VSI(%) = 100 × *W*_*v*_/*W*,

HSI(%) = 100 × *W*_l_/*W*,

*K* (g/cm^3^) = 100 × *W*/*L*^3^,where FBW is the final body weight (g); IBW is the initial body weight (g); *W*_*f*_ is the feed intake (g); *N*_*t*_ is the final number of fish; *N*_0_ is the initial number of fish; *W*_l_ is the liver weight (g); *W*_*v*_ is the viscera weight (g); *W* is the body weight (g); and *L* is the body length (cm).

#### 2.5.2. Proximate Composition of Diets and Whole Body

Proximate composition of diets and whole body was analyzed according to the Association of Official Analytical Chemists (AOAC) method. Moisture was determined by oven-drying at 105°C to constant weight. Crude ash was measured by burning at 550°C for 6 h in a muffle furnace (SXL-1008 muffle furnace, Shanghai Jinghong Experimental Equipment Company). Crude protein was measured by the Kjeldahl method (2300 Auto analyzer, FOSS Tecator, AB, Hoganas, Sweden), and crude lipid was determined by chloroform-methanol extraction method.

#### 2.5.3. Intestinal Histology

The intestine tissue was dehydrated in a series of alcohol solutions and embedded in paraffin. Then, sections (5-6 *μ*m) were cut by semi-automatic slicer (LEICA RM2145), patched, baked, dewaxed, rehydrated, stained with hematoxylin-eosin (H.E), and finally sealed with neutral gum. Sections were observed and photographed with an imaging microscope (Nikon 55I, Tokyo, Japan), and the intestinal villus height, width, and muscle thickness were measured.

#### 2.5.4. Intestinal Digestive Enzyme Activity

Intestinal sample was homogenized with nine times volume of saline (8.6 g/L NaCl) for 1 min and then centrifuged at 3000 r/min (4°C) for 10 min. The supernatant was collected to analyze enzyme activity. The kits used for the measurement of lipase activity and amylase activity were provided by Nanjing Jiancheng Bioengineering Institute (Nanjing, China).

#### 2.5.5. Serum Biochemical Indicators

D-lactic acid (D-LA) was determined by visible spectrophotometric method with kit provided by Shanghai Haling Biotechnology Co., Ltd. Superoxide dismutase (SOD) activity was measured by the xanthine oxidase method. The activities of alkaline phosphatase (AKP), acid phosphatase (ACP), diamine oxidase (DAO), and lysozyme (LZM) were assayed by p-nitrophenyl phosphate method, visible spectrophotometric method, spectrophotometric method, and turbidimetric method, respectively. The corresponding kits were provided by Nanjing Jiancheng Bioengineering Institute (Nanjing, China).

#### 2.5.6. Nutrient Apparent Digestibility and Retention Coefficient

The yttrium contents in diet and feces were analyzed by inductivity-coupled plasma optical emission spectroscopy (ICP-OES; Vista MPX; VARIAN). The apparent digestibility coefficient of dry matter (ADCD) and crude protein (ADCP), protein retention (PR), and lipid retention (LR) were calculated according to the following formulas:

ADCD(%) = 100 × (1 − dietary Y_2_O_3_/feces Y_2_O_3_),

ADCP(%) = 100 × [1 − (dietary Y_2_O_3_ × feces CP)/(feces Y_2_O_3_ × dietary CP)],

PR(%) = 100 × [(final body weight × crude protein of final fish − initial body weight × crude protein of initial fish)/protein intake],

LR(%) = 100 × [(final body weight × crude lipid of final fish − initial body weight × crude lipid of initial fish)/lipid intake].

#### 2.5.7. Intestinal Microbiota

Total DNA from hindgut microbiota was extracted according to the instructions of the E.Z.N.A.® soil kit (Omega BioTek, Norcross, GA, USA). DNA concentration and purity were measured by NanoDrop2000, and DNA extraction quality was measured by 1% agarose gel electrophoresis. PCR amplification of the V3-V4 variable region was performed with primers 338F (5′-ACTCCTACGGGAGGCAGCAG-3′) and 806R (5′-GGACTACHVGGGTWTCTAAT-3′).

Illumina Miseq sequencing was performed using 2% agarose gels for PCR product recovery, and purification was conducted using AxyPrep DNA Gel Extraction Kit (Axygen Biosciences, Union City, CA, USA). Then, elution, detection, and quantification were performed with tris-HCl, 2% agarose electrophoresis, and QuantiFluor™-ST (Promega, USA), respectively. The construction of high-throughput sequencing libraries and sequencing based on the Illumina Miseq platform was performed by Majorbio Bio-Pharm Technology Co., Ltd. Alpha diversity indices including Sobs, Chao, Shannon index, and Good's coverage were calculated with Mothur v1.30.1 (http://www.mothur.org/wiki/Calculators).

#### 2.5.8. Statistical Analysis

The data were expressed as mean ± standard deviation (mean ± SD) and statistically analyzed by SPSS 26.0 software using two-way ANOVA, and multiple comparisons were performed by Tukey method. *P* < 0.05 was the criterion for significant differences.

## 3. Results

### 3.1. Growth Performance and Body Indices

As shown in Table 2, dietary fishmeal level significantly affects the FBW, WG, and *K* value of rainbow trout (*P* < 0.05), while sodium butyrate level and their interaction did not (*P* > 0.05). Low fishmeal group showed significantly lower WG and higher FCR than the high fishmeal group (*P* < 0.05). The addition of sodium butyrate to high or low fishmeal diets had no significant effects on WG and FCR of rainbow trout (*P* > 0.05). There was no significant difference in VSI, HSI, and *K* value among the groups (*P* > 0.05).

### 3.2. Whole Body Composition

In Table 3, no significant differences are found in moisture, ash, crude fat, and crude protein contents among all the groups (*P* > 0.05). There were no significant effects of dietary fishmeal level, sodium butyrate level, and their interaction on whole body composition (*P* > 0.05).

### 3.3. Apparent Digestibility and Nutrients Retention

As shown in Table 4, dietary fishmeal level and sodium butyrate level had no significant effects on the ADCD, ADCP, PR, and LR of rainbow trout, and no significant difference is observed among all the groups (*P* > 0.05).

### 3.4. Digestive Enzyme Activity

The digestive enzyme activities in intestine are shown in Table 5. Dietary fishmeal level did not significantly affect lipase activity (*P* > 0.05), while sodium butyrate level and their interaction did (*P* < 0.05). Dietary fishmeal level, sodium butyrate level, and their interaction significantly affected intestinal amylase (*P* < 0.05). The activities of amylase and lipase were significantly higher in the low fishmeal group than in the high fishmeal group (*P* < 0.05). The addition of 1.0 g/kg and 2.0 g/kg sodium butyrate to the low fishmeal diet significantly decreased the activities of lipase and amylase (*P* < 0.05), but in high fishmeal diet, lipase activity was decreased by 1.0 g/kg sodium butyrate addition, and amylase activity was decreased by 2.0 g/kg sodium butyrate addition (*P* < 0.05).

### 3.5. Serum Biochemical Indices

Dietary fishmeal level and sodium butyrate level significantly affected D-LA level (*P* < 0.05). D-LA was significantly lower in the low fishmeal group than in the high fishmeal group (*P* < 0.05). The addition of sodium butyrate to the high fishmeal diet had no significant effect on the D-LA level (*P* > 0.05), while the addition of 2.0 g/kg sodium butyrate to the low fishmeal diet significantly increased the D-LA level (*P* < 0.05). The addition of 1.0 g/kg sodium butyrate to the low fishmeal diet significantly decreased the activity of DAO and tended to increase the activity of LZM. No significant differences were found in serum SOD, AKP, and ACP activities among all the groups (*P* > 0.05) (Table 6).

### 3.6. Intestinal Morphology

**Figure 1:**
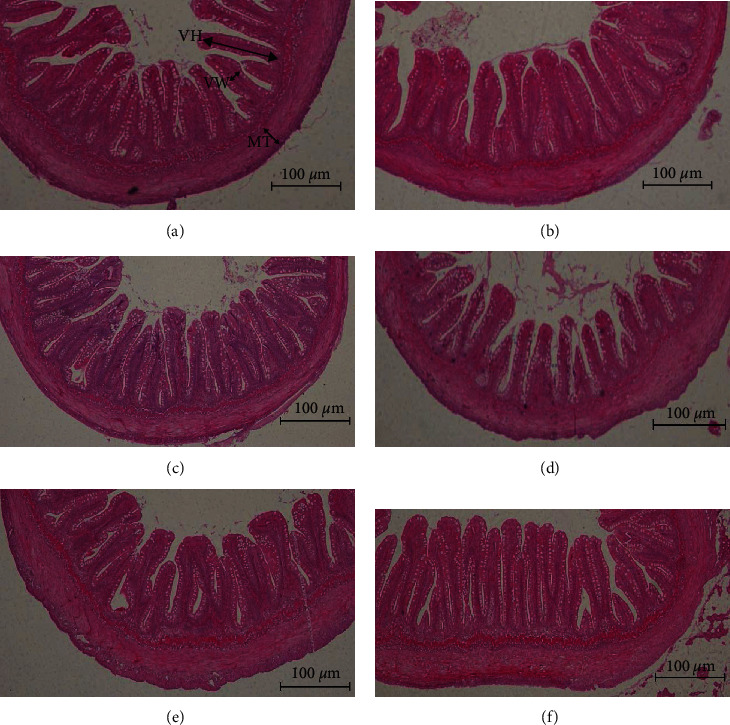
Effect of dietary inclusion of sodium butyrate on intestinal structure of rainbow trout (MT: muscular thickness; VH: villus height; VW: villus width). (a) High fishmeal group; (b) high fishmeal +1.0 g/kg NaB; (c) high fishmeal +2.0 g/kg NaB; (d) low fishmeal group; (e) low fishmeal +1.0 g/kg NaB; and (f) low fishmeal +2.0 g/kg NaB.

The intestinal morphology is shown in Table 7 and Figure 1. Dietary sodium butyrate and the interaction of fishmeal and sodium butyrate significantly affected intestinal villus height (*P* < 0.05). The addition of 1.0 and 2.0 g/kg sodium butyrate to high fishmeal diets and 2.0 g/kg sodium butyrate to low fishmeal diet significantly increased intestinal villus height (*P* < 0.05). Dietary fishmeal, sodium butyrate, and their interaction significantly affected intestinal villus width (*P* < 0.05). The low fishmeal group showed significantly lower muscle thickness than the high fishmeal group (*P* < 0.05), and the addition of 1.0 g/kg sodium butyrate to the low fishmeal diet significantly increased the muscle thickness (*P* < 0.05).

### 3.7. Intestinal Microbial Community

The addition of 1.0 or 2.0 g/kg sodium butyrate to diet did not promote the growth performance; it may be due to the low addition of sodium butyrate. Therefore, intestinal microbial analysis was performed on the 2.0 g/kg sodium butyrate-treated group. As shown in Table 8, the coverage index of each group is close to 1, which indicated that the bacterial community had been fully sampled and the data could represent the bacterial population. The addition of 2.0 g/kg sodium butyrate to the high and low fishmeal diets increased the Sobs and Chao index, indicating that the species richness of intestinal microbiota was significantly increased.

**Figure 2:**
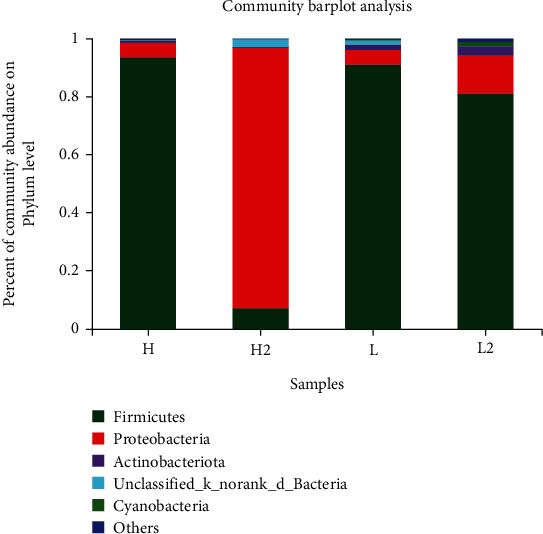
Intestinal microbiota community abundance of rainbow trout at phylum level. H:, High fishmeal group; H2:, High fishmeal + 2.0 g/kg NaB; L:, Low fishmeal group; L2:, Low fishmeal + 2.0 g/kg NaB.

**Figure 3:**
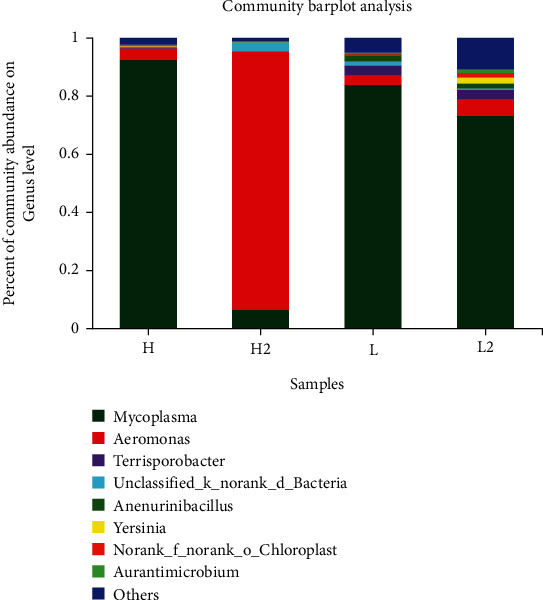
Intestinal microbiota community abundance of rainbow trout at genus level. H: high fishmeal group; H2; high fishmeal +2.0 g/kg NaB, L: low fishmeal group; L2: low fishmeal +2.0 g/kg NaB.

The composition and diversity of intestinal microbiota at the phylum and genus levels are shown in Figures 2 and 3. At the phylum level, the major intestinal microbiota was *Firmicutes*, *Proteobacteria*, *Actinobacteria*, and *Cyanobacteria*. In the high fishmeal group, the dominant phylum was *Firmicutes* accounting for 93.64%, which was changed to *Proteobacteria* (89.83%) when 2.0 g/kg sodium butyrate was added. In the low fishmeal group, the dominant phylum was also *Firmicutes* (90.95%), and the addition of 2.0 g/kg sodium butyrate decreased the abundance of *Firmicutes* to 80.98% and increased the abundance of *Proteobacteria* from 5.14 to 13.02%.

At the genus level, intestine microbial mainly included *Mycoplasma* and *Aeromonas*. In the high fishmeal group, *Mycoplasma* accounted for 92.51%, followed by *Aeromonas* with 3.86%. When 2.0 g/kg sodium butyrate was added in high fishmeal diet, the dominant genus was changed to *Aeromonas* (89.20%). In low fishmeal diet, the abundance of *Mycoplasma* was decreased from 83.83 to 73.28%, and *Aeromonas* was increased from 3.30 to 5.46% by the addition of 2.0 g/kg sodium butyrate.

## 4. Discussion

### 4.1. Effect of Dietary Fishmeal and Sodium Butyrate on Growth Performance of Rainbow Trout

Soybean meal is one of the best plant protein sources to replace fishmeal, but higher levels of fishmeal replacement always imposed a negative influence on fish [22]. In this study, the replacement of 100 g/kg fishmeal with soybean meal (low fish meal diet) significantly decreased the growth performance and decreased feed conversion ratio of rainbow trout, which was consistent with the results in large yellow croaker (*Larimichtys crocea*) [23] and Japanese flounder (*Paralichthy solivaceus*) [24]. The presence of antinutritional factors in soybean meal may be associated with such results. Saponins and lectins can damage the intestinal morphology of fish, while protease inhibitors, *β*-conglycinin, and the imbalanced amino acids in soybean meal also adversely affect the nutrients digestion and absorption [25]. In addition, reduced fishmeal inclusion also leads to the decrease of active components as taurine and unknown growth factors [26].

As the active component in sodium butyrate, butyric acid can promote the absorption of protein and minerals and provide energy to the intestinal epithelial cells for proliferation and differentiation. The dietary supplementation of 1.5 g/kg sodium butyrate nanoparticles significantly improved the growth and feed consumption of tilapia [27]. Silva et al. [28] reported that the feed efficiency and survival of Pacific white shrimp (*Litopenaeus vannamei*) were improved by adding 0.5% and 2.0% sodium butyrate to diets. The supplementation of microencapsulated sodium butyrate in diets with or without oxidized fish oil inclusion significantly increased the weight gain and decreased the feed conversion ratio of juvenile carp (*Cyprinus carpio*) [29]. However, dietary addition of sodium butyrate (0.2%) did not produce significant effects on the growth performance of European sea bass (*Dicentrarchus labrax*) [30, 31]. The supplementation of 0.5% or 2.0% short-chain fatty acids (salts) mixture (acetate: propionic acid: sodium butyrate = 5 : 5 : 2) [21] and the supplementation of a mixture of 10 g/kg sodium formate and sodium butyrate (2 : 1) [20] also did not improve the growth performance of Atlantic salmon and rainbow trout, respectively. In this study, the addition of sodium butyrate in high or low fishmeal diets showed no significant effects on weight gain, feed conversion ratio, and apparent digestibility of dry matter and crude protein. Maybe the dietary effects of sodium butyrate on fishes are related to the feeding habits. Carnivorous, omnivorous, and herbivorous fish may have different responses to dietary sodium butyrate, or cold water fish are not as sensitive to dietary sodium butyrate as warm fish [7]. The hypothesis needs to be further studied in the future.

### 4.2. Effect of Dietary Fishmeal and Sodium Butyrate on the Digestive Enzyme Activities of Rainbow Trout

Digestive enzyme activities are associated with digestive and absorptive capacities, which are crucial to the growth of fish [32]. Results of the present study showed that 50% replacement of fishmeal by soybean meal significantly increased the activities of amylase and lipase, which was similar to the result found in goldfish (*Carassius auratus*) [33]. Nevertheless, high levels of soybean meal substitution for fishmeal in feed caused negative effects on the digestive enzyme activities of fish. For example, 60% and 75% replacement of fishmeal by soybean significantly decreased the digestive enzyme activities of Chinese sucker (*Myxocyprinus asiaticus*) [34] and tilapia [35], respectively. In this study, the addition of sodium butyrate to the low and high fishmeal diet decreased the activities of lipase and amylase. However, the addition of 2.0 g/kg sodium butyrate to diets increased the digestive enzyme activities of golden pompano [5]. Zhao et al. [36] found that sodium butyrate supplementation (1.0 g/kg) increased intestinal lipase activity of yellow catfish (*Pelteobagrus fulvidraco*). Furthermore, in rice field eel (*Monopterus albus*), the digestive enzyme activities were not affected by dietary sodium butyrate [37]. The different results may be associated with the forms and concentrations of sodium butyrate, the structure of digestive organs, and the experimental environment [36].

### 4.3. Effect of Dietary Fishmeal and Sodium Butyrate on the Intestinal Histology of Rainbow Trout

The intestine is the main digestive and absorption organ of fish, and intestinal epithelial cells and intestinal histology play important roles in the digestion and absorption of nutrients [38]. In the current study, the replacement of 50% fishmeal by soybean meal resulted in significant reduction of muscular thickness and disrupted the intestinal mucosa morphology integrity. Zhang et al. [39] reported that replacing 50% or 75% of fishmeal with soybean meal resulted in significant reduction of muscular thickness of Japanese sea bass (*Lateolabrax japonicus*). Similar findings have also been reported in orange-spotted grouper (*Epinephelus coioides*) [40]. ANFs in high soybean meal diets are one of the main reasons for the negative impacts on intestinal structure [24].

Sodium butyrate is a direct source of energy for intestinal epithelial cells, which can promote intestinal cell proliferation, development, and repair mucosal damage. Butyrate can also stimulate the absorption of water and sodium ions in the colon, induce the synthesis of intracellular mRNA and protein, and accelerate the proliferation of intestinal villus [41]. Dietary sodium butyrate was found to increase the intestinal villus height and density [42]. Wu et al. [19] reported that dietary sodium butyrate (0.15%) increased the intestinal muscle thickness and villus height of yellow drum. In juvenile black sea bream, the highest foregut villi height was observed in fish fed with 0.2% microencapsulated sodium butyrate-supplemented diets [10]. In this study, the intestinal villus height in high fishmeal group and the intestinal muscle thickness in low fishmeal group were significantly increased by the addition of sodium butyrate, but both viscerosomatic and hepatosomatic indexes were not significantly altered, which was similar to the study on gilthead sea bream [43]. Meanwhile, the improvement in intestinal histology did not lead to an increase in dry matter and crude protein digestibility, which deserves further investigation.

D-lactic acid (D-LA) and diamine oxidase (DAO) are the metabolic product of bacteria and the enzymes in intestinal epithelial cells, respectively, but they do not participate in metabolism when entering the body. If intestinal mucosal cells are damaged, intestinal mucosal permeability will increase, and intestinal bacteria and epithelial cells will produce large amounts of D-LA and DAO, respectively, which enter the blood through damaged mucosal cells, leading to the increase of D-LA level and DAO activity in blood. Thus, the serum change of D-LA level and DAO activity can reflect the permeability of intestinal mucosal and the function of intestinal mucosal barrier [39]. In this study, the addition of 1.0 g/kg sodium butyrate to the low fishmeal diet significantly decreased the serum DAO activity, which meant the intestinal mucosal permeability was decreased, indicating that sodium butyrate could repair the mucosal barrier. Meanwhile, no significant change in serum D-LA level was found when sodium butyrate was added to the high fishmeal diet, but the addition of sodium butyrate to the low fishmeal diet tended to increase the D-LA level. The reason is not clear and needs further study.

### 4.4. Effect of Dietary Fishmeal and Butyrate on the Intestinal Microbial Community of Rainbow Trout

As an important part of the intestinal tract, intestinal microbial community can promote the digestion and absorption of nutrients and induce mucosal immune protection. A stable microbial community is a guarantee to intestinal health of fish. Long-term exposure of intestine to feeds and microbiota, together with the changes in the internal and external environment, can change the intestinal microbial community [44, 45]. The present study indicated that the replacement of fishmeal with soybean meal had no effect on the species of the dominant phylum (*Firmicutes* and *Proteobacteria*) in the intestine of rainbow trout, but changed the intestinal microbiota composition and increased the abundance of *Proteobacteria*. Desai et al. [46] also found that plant diets increased the ratio of *Firmicutes* to *Proteobacteria* in intestinal microbiota of rainbow trout and enhanced the overall abundance and diversity of intestinal microbiota. At present, few studies have been conducted on the effect of sodium butyrate on intestinal microbiota of fish. Piazzon et al. [47] found that the relative abundance of *Firmicutes*, *Bacteroidetes*, and *Fusobacteria* in the intestinal microbial community increased 139 times, 8.5 times, and 23.2 times, respectively, when 0.4% sodium butyrate preparation (70% effective content) was added to the diet of sea bream. In turbot, the addition of 0.2% sodium butyrate to high soybean meal diet significantly increased the relative abundance of *Proteobacteria*, *Deinococcus-Thermus*, and *Actinobacteria* and decreased the relative abundance of *Bacteroidetes* and *Deferribacteria* [17]. In this trial, the addition of 2.0 g/kg sodium butyrate to high and low fishmeal diets decreased the abundance of *Firmicutes* and increased the abundance of *Proteobacteria* in intestine. However, the role of *Firmicutes* and *Proteobacteria* in the intestine of rainbow trout is still unclear and needs to be further investigated.

## 5. Conclusion

Under the present conditions, the addition of sodium butyrate to diet containing 200 or 100 g/kg fishmeal did not promote the growth performance and feed utilization of rainbow trout, but improved the intestinal morphology and changed the intestinal microbial composition.

## Figures and Tables

**Table 1:** The formulation and proximate composition of experimental diets (air dry basis, g/kg).

Ingredients	NaB supplementation in high fishmeal diet			NaB supplementation in low fishmeal diet		
	0.0	1.0	2.0	0.0	1.0	2.0
Fish meal	200.0	200.0	200.0	100.0	100.0	100.0
Soy bean meal	50.0	50.0	50.0	225.0	225.0	225.0
Soy protein concentrate	100.0	100.0	100.0	100.0	100.0	100.0
Wheat flour	259.5	258.5	257.5	175.9	174.9	173.9
Corn gluten meal	50.0	50.0	50.0	50.0	50.0	50.0
Cottonseed protein concentrate	100.0	100.0	100.0	100.0	100.0	100.0
Meat and bone meal	90.0	90.0	90.0	90.0	90.0	90.0
Brewers dried yeast	50.0	50.0	50.0	50.0.	50.0	50.0
Fish oil	30.0	30.0	30.0	37.0	37.0	37.0
Soybean oil	30.0	30.0	30.0	30.0	30.0	30.0
DL-methionine	0.0	0.0	0.0	0.9	0.9	0.9
L-Lysine	0.0	0.0	0.0	0.7	0.7	0.7
Vitamin premix^a^	10.0	10.0	10.0	10.0	10.0	10.0
Mineral premix^b^	30.0	30.0	30.0	30.0	30.0	30.0
Y_2_O_3_	0.5	0.5	0.5	0.5	0.5	0.5
Total	1000.0	1000.0	1000.0	1000.0	1000.0	1000.0
Proximate composition						
Crude protein	441.9	437.5	438.6	434.0	435.8	436.0
Crude lipid	85.4	81.5	77.9	82.0	82.2	81.6
Crude ash	133.9	135.1	134.2	128.6	130.8	131.1
Moisture	63.6	65.2	60.3	62.1	63.7	60.9

^a^Vitamin premix (mg or IU/kg diet): VA, 10000IU; VD_3_, 3000IU; VE, 150IU; VK_3_, 12.17 mg; VB_1_, 20 mg; VB_2_, 20 mg; VB_3_, 100 mg; VB_6_, 22 mg; VB_12_, 0.15 mg; VC, 1000 mg; biotin, 0.6 mg; folic acid, 8 mg; inositol, 500 mg. ^b^Mineral premix (mg/kg diet): iodine, 1.5 mg; cobalt, 0.6 mg; copper, 3 mg; iron, 63 mg; zinc, 89 mg; manganese, 11.45 mg; selenium, 0.24 mg; magnesium, 180 mg.

**Table 2:** Effect of dietary sodium butyrate on the growth performance of rainbow trout.

Items	NaB supplementation in high fishmeal diet g/kg			NaB supplementation in low fishmeal diet g/kg			Two-way ANOVA		
	0.0	1.0	2.0	0.0	1.0	2.0	*P*	*S*	*P* × *S*
IBW/g	33.00 ± 0.10	33.00 ± 0.10	32.90 ± 0.2	33.00 ± 0.10	33.00 ± 0.10	33.00 ± 0.10			
FBW/g	158.9 ± 1.8^a^	153.5 ± 3.4^ab^	152.6 ± 3.3^abc^	149.2 ± 3.3^bc^	144.5 ± 5.2^c^	149.1 ± 6.8^bc^	0.003	0.154	0.407
WG/%	381.7 ± 5.4^a^	365.2 ± 10.4^ab^	362.4 ± 10.1^ab^	352.1 ± 10.0^bc^	337.7 ± 15.7^c^	351.8 ± 20.6^bc^	0.003	0.154	0.407
FCR	1.04 ± 0.01^a^	1.08 ± 0.03^ab^	1.09 ± 0.03^abc^	1.12 ± 0.03^bc^	1.17 ± 0.06^c^	1.13 ± 0.07^bc^	0.004	0.181	0.412
SR/%	100.0 ± 0.00	100.0 ± 0.00	100.0 ± 0.00	100.0 ± 0.00	100.0 ± 0.00	100.0 ± 0.00			
*K*/(g/cm^3^)	1.46 ± 0.07	1.43 ± 0.05	1.38 ± 0.11	1.44 ± 0.09	1.43 ± 0.09	1.40 ± 0.09	0.837	0.092	0.731
VSI/%	7.93 ± 0.70	7.83 ± 0.62	8.01 ± 0.73	7.59 ± 0.57	8.29 ± 1.00	8.41 ± 1.23	0.441	0.262	0.292
HSI/%	0.99 ± 0.00	0.93 ± 0.02	1.00 ± 0.05	0.95 ± 0.07	0.93 ± 0.07	0.90 ± 0.04	0.096	0.438	0.255

IBW: initial body weight; FBW: final body weight; WG: weight gain; FCR: feed conversion ratio; SR: survival ratio; *K*: condition factor; VSI: viscerosomatic index; HSI: hepatosomatic index. In the same row, values without letter or with the same letter superscripts mean no significant difference (*P* > 0.05), while with different small letter superscripts mean significant difference (*P* < 0.05). *P*: dietary fishmeal level; *S*: sodium butyrate level. The same as the following tables.

**Table 3:** Effect of dietary sodium butyrate on whole body proximate composition of rainbow trout (g/kg, fresh weight).

Items	NaB supplementation in high fishmeal diet g/kg			NaB supplementation in low fishmeal diet g/kg			Two-way ANOVA		
	0.0	1.0	2.0	0.0	1.0	2.0	*P*	*S*	*P* × *S*
Crude protein	175.5 ± 0.5	172.2 ± 6.0	173.4 ± 3.9	171.9 ± 3.9	173.9 ± 3.6	174.5 ± 8.3	0.915	0.953	0.672
Crude lipid	23.02 ± 0.56	23.41 ± 0.85	22.85 ± 0.68	23.01 ± 1.63	23.65 ± 1.72	24.25 ± 0.57	0.322	0.652	0.522
Crude ash	23.67 ± 1.56	23.67 ± 1.27	22.10 ± 0.42	24.17 ± 1.10	23.80 ± 0.80	24.30 ± 1.73	0.160	0.654	0.412
Moisture	720.2 ± 4.8	719.6 ± 6.0	712.3 ± 3.2	722.8 ± 6.2	720.7 ± 4.2	718.0 ± 8.6	0.269	0.180	0.787

**Table 4:** Effect of dietary sodium butyrate on nutrient utilization of rainbow trout.

Items	NaB supplementation in high fishmeal diet			NaB supplementation in low fishmeal diet			Two-way ANOVA		
	0.0	1.0	2.0	0.0	1.0	2.0	*P*	*S*	*P* × *S*
ADCD/%	77.36 ± 0.89	76.10 ± 1.66	76.24 ± 1.05	76.66 ± 0.64	75.26 ± 1.74	75.28 ± 1.56	0.208	0.192	0.986
ADCP/%	93.99 ± 0.36	94.38 ± 0.70	94.25 ± 0.63	94.86 ± 0.71	94.41 ± 0.28	94.66 ± 0.53	0.125	0.983	0.458
PR/%	41.88 ± 0.91	39.50 ± 2.84	39.22 ± 1.17	38.17 ± 0.76	38.30 ± 1.49	38.63 ± 2.75	0.095	0.600	0.434
LR/%	30.60 ± 0.55	31.45 ± 1.5	31.56 ± 0.36	31.41 ± 0.27	31.50 ± 0.18	31.49 ± 2.40	0.701	0.788	0.846

**Table 5:** Effect of dietary sodium butyrate on digestive enzyme activities in foregut of rainbow trout.

Items	NaB supplementation in high fishmeal diet			NaB supplementation in low fishmeal diet			Two-way ANOVA		
	0.0	1.0	2.0	0.0	1.0	2.0	*P*	*S*	*P* × *S*
Lipase (U/gprot)	0.62 ± 0.05^ab^	0.50 ± 0.03^cd^	0.60 ± 0.01^ab^	0.69 ± 0.01^a^	0.53 ± 0.02^bc^	0.43 ± 0.01^d^	0.185	0.000	0.001
Amylase (U/gprot)	55.53 ± 10.87^bc^	50.78 ± 7.62^bc^	22.98 ± 1.94^d^	96.84 ± 5.71^a^	25.80 ± 3.65^d^	57.87 ± 7.88^b^	0.005	0.000	0.001

**Table 6:** Effect of dietary sodium butyrate on serum biochemical indices of rainbow trout.

Items	NaB supplementation in high fishmeal diet			NaB supplementation in low fishmeal diet			Two-way ANOVA		
	0.0	1.0	2.0	0.0	1.0	2.0	*P*	*S*	*P* × *S*
D-LA (*μ*mol/mL)	11.08 ± 0.84^a^	11.45 ± 0.08^a^	11.28 ± 0.32^a^	9.48 ± 0.56^b^	10.66 ± 0.08^ab^	11.65 ± 0.20^a^	0.038	0.024	0.052
SOD (U/mL)	108.6 ± 13.1	106.9 ± 9.6	112.6 ± 1.9	101.56 ± 6.5	107.1 ± 6.5	104.3 ± 4.4	0.289	0.831	0.720
AKP (U/L)	6.86 ± 1.17	6.24 ± 0.54	7.24 ± 0.40	7.20 ± 1.21	6.67 ± 1.25	6.65 ± 1.25	0.903	0.595	0.656
ACP (U/mL)	0.12 ± 0.01	0.12 ± 0.01	0.11 ± 0.00	0.12 ± 0.01	0.11 ± 0.01	0.12 ± 0.01	0.259	0.434	0.020
DAO (U/L)	11.06 ± 0.40^ab^	11.00 ± 0.74^ab^	11.06 ± 0.81^ab^	11.52 ± 0.20^a^	10.13 ± 0.99^b^	11.52 ± 0.35^a^	0.185	0.953	0.231
LZM (*μ*g/mL)	10.91 ± 0.56^ab^	10.54 ± 0.77^a^	11.76 ± 0.00^ab^	11.40 ± 1.04^ab^	12.01 ± 0.93^b^	11.40 ± 1.04^ab^	0.197	0.674	0.187

**Table 7:** Effect of dietary sodium butyrate on intestinal histology of rainbow trout.

Items	NaB supplementation in high fishmeal diet			NaB supplementation in low fishmeal diet			Two-way ANOVA		
	0.0	1.0	2.0	0.0	1.0	2.0	*P*	*S*	*P* × *S*
Muscle thickness/*μ*m	161.4 ± 21.9^ab^	150.9 ± 8.1^abc^	141.7 ± 12.6^bc^	132.9 ± 8.5^c^	160.3 ± 7.9^ab^	167.1 ± 5.9^a^	0.716	0.446	0.005
Villus height/*μ*m	589.9 ± 19.9^c^	693.5 ± 21.4^ab^	656.8 ± 52.1^b^	597.4 ± 36.2^c^	554.8 ± 30.3^c^	717.4 ± 17.6^a^	0.094	0.000	0.000
Villus width/*μ*m	108.3 ± 3.7*b*	110.8 ± 5.3^b^	114.5 ± 8.5^b^	112.9 ± 11.0^b^	152.9 ± 6.7^a^	121.1 ± 10.1^b^	0.000	0.001	0.001

**Table 8:** Intestinal microbial diversity index of rainbow trout.

Items	NaB supplementation in high fishmeal diet		NaB supplementation in low fishmeal diet		Two-way ANOVA		
	0.0	2.0	0.0	2.0	*P*	*S*	*P* × *S*
Sobs	127.4 ± 42.8^b^	146.3 ± 12.4^b^	245.5 ± 74.2^ab^	307.8 ± 111.8^a^	0.003	0.299	0.572
Chao	157.3 ± 55.4^b^	216.2 ± 49.7^b^	295.9 ± 103.6^ab^	367.0 ± 117.7^a^	0.008	0.170	0.893
Shannon	0.42 ± 0.23^b^	0.53 ± 0.40^b^	0.92 ± 0.01^ab^	1.50 ± 0.86^a^	0.023	0.248	0.419
Coverage	0.999	0.999	0.999	0.999	0.052	0.512	0.941

Sobs: the observed richness; Chao: the Chao1 estimator; Shannon: the Shannon diversity index; Coverage: the Good's coverage.

## Data Availability

All data generated or analyzed during this study are included in this article.

## References

[1] China Fishery Statistical Yearbook (2021). *Beijing*.

[2] Tacon A. G. J., Metian M. (2008). Global overview on the use of fish meal and fish oil in industrially compounded aquafeeds: trends and future prospects. *Aquaculture*.

[3] Deng J., Mai K. S., Ai Q. H. (2006). Effects of replacing fish meal with soy protein concentrate on feed intake and growth of juvenile Japanese flounder, *Paralichthys olivaceus*. *Aquaculture*.

[4] Krogdahl A., Bakke-Mckellep A. M., Roed K. H., Baeverfjord G. (2000). Feeding Atlantic salmon Salmo salar L. soybean products: effects on disease resistance (furunculosis), and lysozyme and IgM levels in the intestinal mucosa. *Aquaculture Nutrition*.

[5] Zhou J. S., Guo P., Yu H. B., Ji H., Lai Z. W., Chen Y. A. (2019). Growth performance, lipid metabolism, and health status of grass carp (*Ctenopharyngodon idella*) fed three different forms of sodium butyrate. *Fish Physiology and Biochemistry*.

[6] Hoseinifar S. H., Sun Y. Z., Caipang C. M. (2017). Short-chain fatty acids as feed supplements for sustainable aquaculture: an updated view. *Aquaculture Research*.

[7] Tran N. T., Li Z. Z., Wang S. Q. (2020). Progress and perspectives of short-chain fatty acids in aquaculture. *Reviews in Aquaculture*.

[8] Robles R., Lozano A. B., Sevilla A., Márquez L., Nuez-Ortín W., Moyano F. J. (2013). Effect of partially protected butyrate used as feed additive on growth and intestinal metabolism in sea bream (*Sparus aurata*). *Fish Physiology and Biochemistry*.

[9] Zhou C. P., Lin H. Z., Huang Z., Wang J., Wang Y., Yu W. (2019). Effect of dietary sodium butyrate on growth performance, enzyme activities and intestinal proliferation-related gene expression of juvenile golden pompano *Trachinotus ovatus*. *Aquaculture Nutrition*.

[10] Ullah S., Zhang G. W., Zhang J. Z. (2020). Effects of microencapsulated sodium butyrate supplementation on growth performance, intestinal development and antioxidative capacity of juvenile black sea bream (*Acanthopagrus schlegelii*). *Aquaculture Research*.

[11] Abd El-Naby A. S., Khattaby A. E.-R. A., Samir F., Awad S. M. M., Abdel-Tawwab M. (2019). Stimulatory effect of dietary butyrate on growth, immune response, and resistance of Nile tilapia, *Oreochromis niloticus* against *Aeromonas hydrophila* infection. *Animal Feed Science and Technology*.

[12] Dawood M. A. O., Eweedah N. M., Elbialy Z. I., Abdelhamid A. I. (2020). Dietary sodium butyrate ameliorated the blood stress biomarkers, heat shock proteins, and immune response of Nile tilapia (*Oreochromis niloticus*) exposed to heat stress. *Journal of Thermal Biology*.

[13] Jesus G. F. A., Pereira S. A., Owatari M. S. (2019). Use of protected forms of sodium butyrate benefit the development and intestinal health of Nile tilapia during the sexual reversion period. *Aquaculture*.

[14] Liu M. M., Guo W., Wu F., Qu Q. C., Tan Q. S., Gong W. B. (2017). Dietary supplementation of sodium butyrate may benefit growth performance and intestinal function in juvenile grass carp (*Ctenopharyngodon idellus*). *Aquaculture Research*.

[15] Tian L., Zhou X. Q., Jiang W. D. (2017). Sodium butyrate improved intestinal immune function associated with NF-kappaB and p38MAPK signalling pathways in young grass carp (*Ctenopharyngodon idella*). *Fish & Shellfish Immunology*.

[16] Wu P., Tian L., Zhou X. Q. (2018). Sodium butyrate enhanced physical barrier function referring to Nrf2, JNK and MLCK signaling pathways in the intestine of young grass carp (*Ctenopharyngodon idella*). *Fish & Shellfish Immunology*.

[17] Liu Y., Chen Z. C., Dai J. H. (2019). Sodium butyrate supplementation in high-soybean meal diets for turbot (*Scophthalmus maximus L.*): effects on inflammatory status, mucosal barriers and microbiota in the intestine. *Fish & Shellfish Immunology*.

[18] Luz J. R., Ramos A. P. S., Melo J. F. B., Braga L. G. T. (2019). Use of sodium butyrate in the feeding of *Arapaima gigas* (Schinz, 1822) juvenile. *Aquaculture*.

[19] Wu X., Wang L. G., Xie Q. P., Tan P. (2020). Effects of dietary sodium butyrate on growth, diet conversion, body chemical compositions and distal intestinal health in yellow drum (*Nibea albiflora*, Richardson). *Aquaculture Research*.

[20] Gao Y. L., Storebakken T., Shearer K. D., Penn M., Øverland M. (2011). Supplementation of fishmeal and plant protein-based diets for rainbow trout with a mixture of sodium formate and butyrate. *Aquaculture*.

[21] Bjerkeng B., Storebakken T., Wathne E. (1999). Cholesterol and short-chain fatty acids in diets for Atlantic salmon (*Salmo salar L.*): effects on growth, organ indices, macronutrient digestibility, and fatty acid composition. *Aquaculture Nutrition*.

[22] Gatlin D. M., Barrows F. T., Brown P. (2007). Expanding the utilization of sustainable plant products in aquafeeds: a review. *Aquaculture Research*.

[23] Wang P., Zhou Q. C., Feng J., He J. J., Lou Y. D., Zhu J. Q. (2019). Effect of dietary fermented soybean meal on growth, intestinal morphology and microbiota in juvenile large yellow croaker, *Larimichthys crocea*. *Aquaculture Research*.

[24] Chen W., Ai Q. H., Mai K. S. (2011). Effects of dietary soybean saponins on feed intake, growth performance, digestibility and intestinal structure in juvenile Japanese flounder (*Paralichthys olivaceus*). *Aquaculture*.

[25] Francis G., Makkar H. P. S., Becker K. (2001). Antinutritional factors present in plant-derived alternate fish feed ingredients and their effects in fish. *Aquaculture*.

[26] Hien T. T. T., Be T. T., Lee C. M., Bengtson D. A. (2015). Development of formulated diets for snakehead (*Channa striata* and *Channa micropeltes*): can phytase and taurine supplementation increase use of soybean meal to replace fish meal?. *Aquaculture*.

[27] Abdel-Tawwab M., Shukry M., Farrag F. A., El-Shafai N. M., Dawood M. A. O., Abdel-Latif H. M. R. (2021). Dietary sodium butyrate nanoparticles enhanced growth, digestive enzyme activities, intestinal histomorphometry, and transcription of growth-related genes in Nile tilapia juveniles. *Aquaculture*.

[28] da Silva B. C., Vieira F. D. N., Mouriño J. L. P., Bolivar N., Seiffert W. Q. (2016). Butyrate and propionate improve the growth performance of *Litopenaeus vannamei*. *Aquaculture Research*.

[29] Liu W. S., Yang Y., Zhang J. L., Gatlin D. M., Ringø E., Zhou Z. Z. (2014). Effects of dietary microencapsulated sodium butyrate on growth, intestinal mucosal morphology, immune response and adhesive bacteria in juvenile common carp (*Cyprinus carpio*) pre-fed with or without oxidised oil. *British Journal Nutrition*.

[30] Rimoldi S., Finzi G., Ceccotti C. (2016). Butyrate and taurine exert a mitigating effect on the inflamed distal intestine of European sea bass fed with a high percentage of soybean meal. *Fisheries and Aquatic Sciences*.

[31] Terova G., Díaz N., Rimoldi S., Ceccotti C., Gliozheni E., Piferrer F. (2016). Effects of sodium butyrate treatment on histone modifications and the expression of genes related to epigenetic regulatory mechanisms and immune response in European sea bass (*Dicentrarchus Labrax*) fed a plant-based diet. *PLoS One*.

[32] Krogdahl A., Bakke-Mckellep A. M. (2005). Fasting and refeeding cause rapid changes in intestinal tissue mass and digestive enzyme capacities of Atlantic salmon (*Salmo salar L.*). *Comparative Biochemistry Biochemistry and Physiology Part A: Molecular & Integrative Physiology*.

[33] Liu X. R., Han B., Xu J. (2020). Replacement of fishmeal with soybean meal affects the growth performance, digestive enzymes, intestinal microbiota and immunity of *Carassius auratus gibelio♀ × Cyprinus carpio♂*. *Aquaculture Reports*.

[34] Yu D. H., Gong S. Y., Yuan Y. C., Lin Y. C. (2013). Effects of replacing fish meal with soybean meal on growth, body composition and digestive enzyme activities of juvenile Chinese sucker, *Myxocyprinus asiaticus*. *Aquaculture Nutrition*.

[35] Lin S. M., Luo L. (2011). Effects of different levels of soybean meal inclusion in replacement for fish meal on growth, digestive enzymes and transaminase activities in practical diets for juvenile tilapia, *Oreochromis niloticus×O. aureus*. *Animal Feed Science and Technology*.

[36] Zhao H. X., Wang G. X., Wang H. R. (2021). Effects of dietary sodium butyrate on growth, digestive enzymes, body composition and nutrient retention-related gene expression of juvenile yellow catfish (*Pelteobagrus fulvidraco*). *Animal Nutrition*.

[37] Zhang J. Z., Zhong L., Chi S. Y., Chu W. Y., Liu Y. L., Hu Y. (2020). Sodium butyrate supplementation in high-soybean meal diets for juvenile rice field eel (*Monopterus albus*): effects on growth, immune response and intestinal health. *Aquaculture*.

[38] Ray A. K., Ghosh K., Ringø E. (2012). Enzyme-producing bacteria isolated from fish gut: a review. *Aquaculture Nutrition*.

[39] Zhang C. X., Rahimnejad S., Wang Y. R. (2018). Substituting fish meal with soybean meal in diets for Japanese seabass (*Lateolabrax japonicus*): effects on growth, digestive enzymes activity, gut histology, and expression of gut inflammatory and transporter genes. *Aquaculture*.

[40] Wang Y. R., Wang L., Zhang C. X., Song K. (2017). Effects of substituting fishmeal with soybean meal on growth performance and intestinal morphology in orange-spotted grouper (*Epinephelus coioides*). *Aquaculture Reports*.

[41] Canani R. B., Costanzo M. D., Leone L. (2012). The epigenetic effects of butyrate: potential therapeutic implications for clinical practice. *Clinical Epigenetics*.

[42] Fang L., Wang Q., Guo X., Pan X., Li X. (2021). Effects of dietary sodium butyrate on growth performance, antioxidant capacity, intestinal histomorphology and immune response in juvenile Pengze crucian carp (*Carassius auratus* Pengze). *Aquaculture Reports*.

[43] Estensoro I., Ballester-Lozano G., Benedito-Palos L. (2016). Dietary butyrate helps to restore the intestinal status of a marine teleost (*Sparus aurata*) fed extreme diets low in fish meal and fish oil. *PLoS One*.

[44] Dehler C. E., Secombes C. J., Martin S. A. M. (2017). Environmental and physiological factors shape the gut microbiota of Atlantic salmon parr (*Salmo salar L.*). *Aquaculture*.

[45] Ringø E., Zhou Z., Vecino J. L. G. (2016). Effect of dietary components on the gut microbiota of aquatic animals. A never-ending story?. *Aquaculture Nutrition*.

[46] Desai A. R., Links M. G., Collins S. A. (2012). Effects of plant-based diets on the distal gut microbiome of rainbow trout (*Oncorhynchus mykiss*). *Aquaculture*.

[47] Piazzon M. C., Calduch-Giner J. A., Fouz B. (2017). Under control: how a dietary additive can restore the gut microbiome and proteomic profile, and improve disease resilience in a marine teleostean fish fed vegetable diets. *Microbiome*.

